# Supplementary material for: The First Large Identification of 3ANX and NX Producing Isolates of Fusarium graminearum in Manitoba, Western Canada
Source: Toxins (Basel). 2025 Jan 17;17(1):45. doi: 10.3390/toxins17010045 (PMC11769337; doi:10.3390/toxins17010045)
Supplement: Supplementary file 1 [file toxins-17-00045-s001.zip › Henriquez et al, Toxins_Supplementary Figure S1_3.pdf]

toxins

Article

The first large identification of 3ANX and NX producing isolates of *Fusarium graminearum* in Western Canada

M.A. Henriquez [1*], S. Sura [1*], S. Walkowiak[2], D. Kaminski[3], A. Kirk[3], M.W. Sumarah[4], P. Santhanam[1], N. Kepeshchuk[1], J. Carlson[1], E. RoTimi Ojo[3], P. de Rocquigny[3,5], H. Derksen[3,6]

[1] Morden Research and Development Centre, Agriculture and Agri-Food Canada, Morden, MB, Canada
[2] Canadian Grain Commission, Grain Research Laboratory, Winnipeg, MB, Canada
[3] Manitoba Agriculture, 65-3rd Avenue NE, Carman, MB, Canada
[4] London Research and Development Center, Agriculture and Agri-Food Canada, London, Canada
[5] Manitoba Crop Alliance, 38-4th Avenue NE, Carman, MB, Canada
[6] UPL AgroSolutions Canada, 2-400 Michener Road, Guelph, ON, Canada
* Correspondence: Maria Antonia Henriquez, MariaAntonia.Henriquez@agr.gc.ca; Srinivas Sura, Srinivas.Sura@agr.gc.ca;

Supplementary Material:

Supplementary Table S1. Total 3ANX (also called NX-2) producers isolates identified worldwide.
Supplementary Table S1 Excel File.

Supplementary Table S2. Location of *F. graminearum* strains used in this study.
Supplementary Table S2 Excel File.

toxins

MDPI

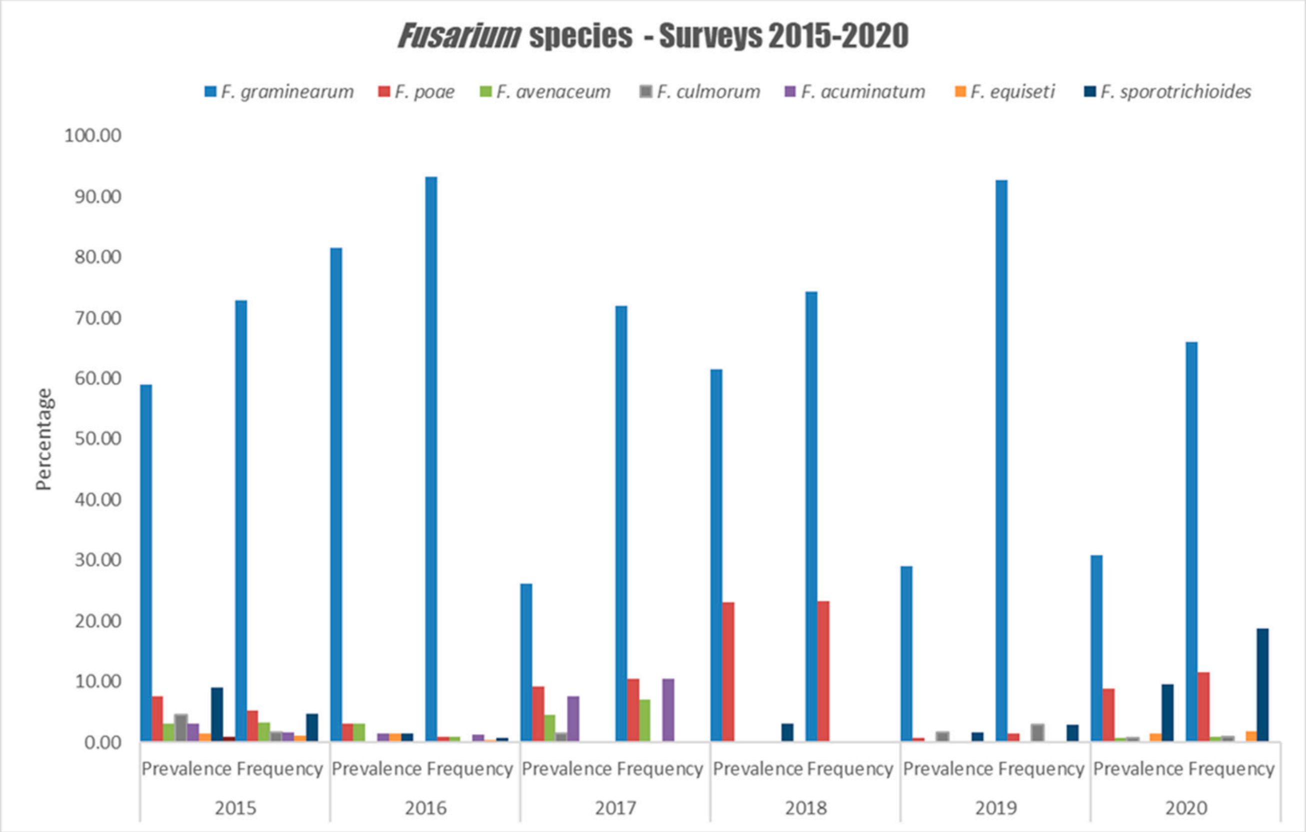

Fusarium Species	2015		2016		2017		2018		2019		2020	
	Prevalence	Frequency	Prevalence	Frequency	Prevalence	Frequency	Prevalence	Frequency	Prevalence	Frequency	Prevalence	Frequency
F. graminearum	59.09	72.87	81.5	93.3	26.2	71.9	61.5	74.4	29.0	92.6	30.9	66.1
F. poae	7.58	5.32	3.1	1.0	9.2	10.5	23.1	23.3	0.8	1.5	8.8	11.6
F. avenaceum	3.03	3.19	3.1	1.0	4.6	7.0	0.0	0.0	0.0	0.0	0.7	0.9
F. culmorum	4.55	1.60	0.0	0.0	1.5	0.0	0.0	0.0	1.6	2.9	0.7	0.9
F. acuminatum	3.03	1.60	1.5	1.3	7.7	10.5	0.0	0.0	0.0	0.0	0.0	0.0
F. equiseti	1.52	1.06	1.5	0.3	0.0	0.0	0.0	0.0	0.0	0.0	1.5	1.8
F. sporotrichioides	9.09	4.79	1.5	0.7	0.0	0.0	3.1	0.0	1.61	2.94	9.6	18.8

Supplementary Figure S1. ***Fusarium*** **species collected from 2015 to 2020.**

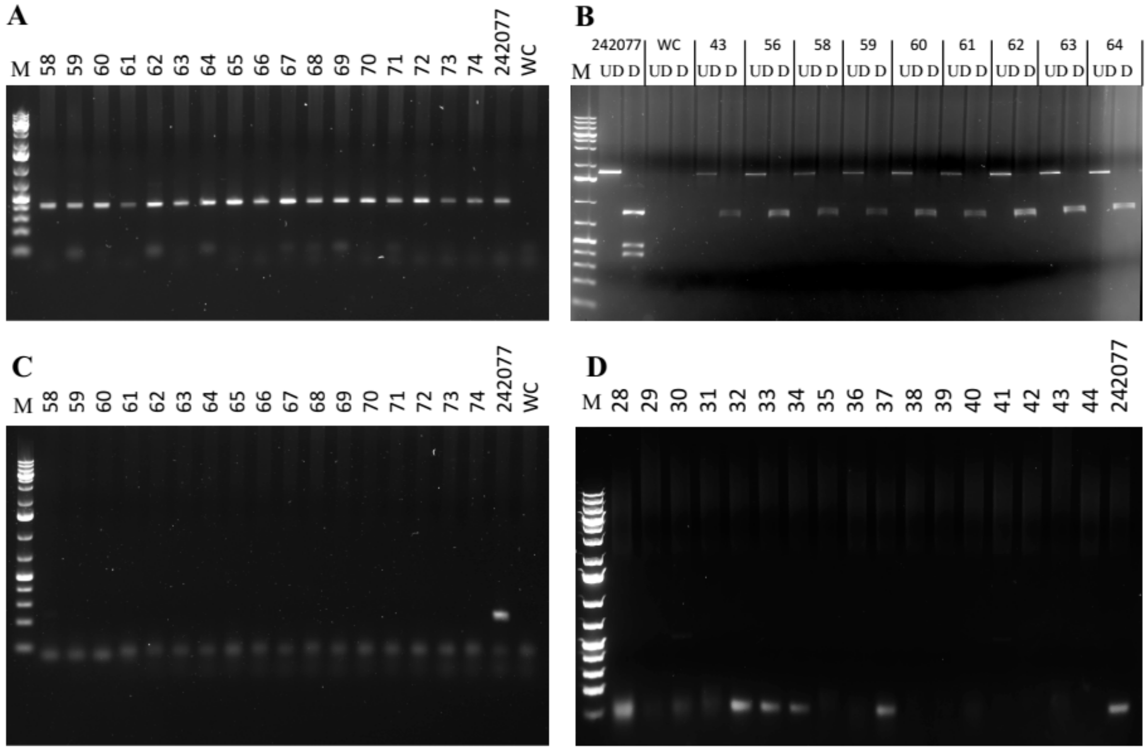

Supplementary Figure S2. Electrophoresis of the PCR products using different primers. (A) *Fusarium graminearum* species-specific PCR using Nicholson et al., 1998 primers; (B) PCR-RFLP assay undigested (UD) and digested (D) PCR products using Liang et al., 2014 primers; (C) PCR products using Toomajian assay (Crippin et al., 2019); (D) The NX-Tri1-F and NX-*Tri1*-R primers used in the current study. Lane M, DNA marker; WC, water control; DAOM 242077 positive 3ANX isolate; Lane numbers (i.e.: 58-74) samples ID from Error! Reference source not found..

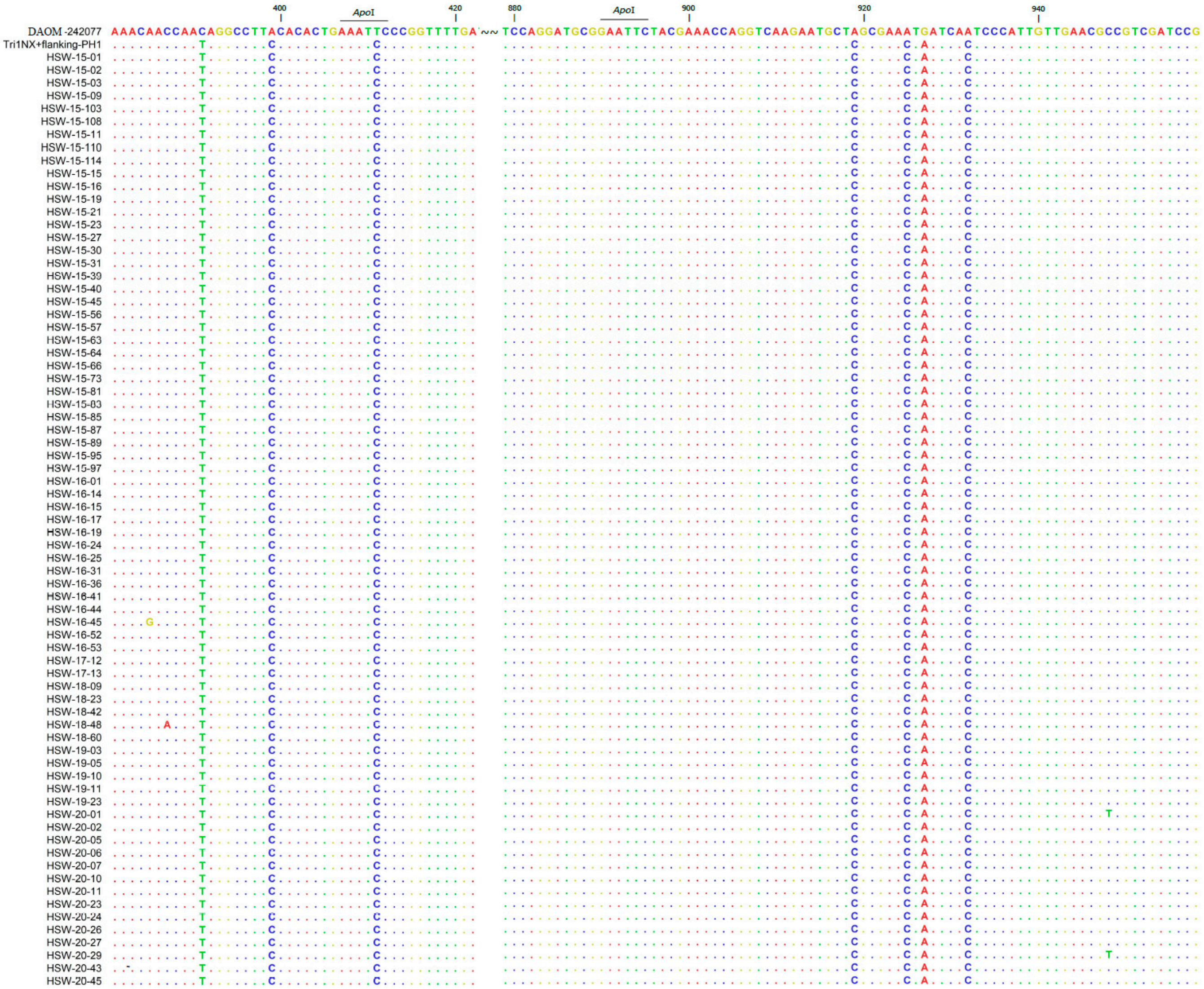

Supplementary Figure S3. Multiple sequence alignment of a region of *Tri1* gene. The *Apo*I site (AAATTC) at 407th nucleotide from the start codon is present only in the isolate DAOM 242077 and in the remaining 73 isolates, *Apo*I site was abolished due to T/C transition. The second *Apo*I site (GAATTC) starting at 889th nucleotide was conserved in all 73 tested isolates.
